# Commentary: Study the Features of 57 Confirmed CRISPR Loci in 38 Strains of *Staphylococcus aureus*

**DOI:** 10.3389/fmicb.2019.00059

**Published:** 2019-02-01

**Authors:** Tingting Mao, Jinzhao Long, Guangcai Duan, Haiyan Yang

**Affiliations:** Department of Epidemiology, College of Public Health of Zhengzhou University, Zhengzhou, China

**Keywords:** CRISPR, *Staphylococcus aureus*, *Staphylococcus argenteus*, bioinformatics, data sources

As mentioned in the article by Xihong Zhao, Zhixue Yu and Zhenbo Xu, bioinformatics analysis provides data support for bacterial typing, traceability analysis, and exploration of clustered regularly interspaced short palindromic repeats (CRISPR) (Zhao et al., 2018). However, there are relatively few studies on the CRISPR of *Staphylococcus aureus*. For example, Yang et al. (2015) analyzed the features of 45 identified CRISPR loci in 32 *S. aureus*. Zhang and Ye (2017) studied the CRISPR like elements in *S. aureus*. These articles have contributed greatly to our understanding of the distribution, architecture, function, and evolution of *S. aureus* CRISPR. At present, the research about the characteristics of CRISPR has made some headway. For instance, based on the specificity of virulence genes and CRISPR, Liu et al. (2011) have developed a sequence typing scheme (designated CRISPR–MVLST) to subtype *Salmonella enterica* isolated from different sources, Shariat et al. (2013) combined the method with multiple site sequence typing to distinguish the outbreak isolates during pathogen outbreaks. Furthermore, with the development of CRISPR-mediated genome editing and engineering, these CRISPR-based tools will have major implications for both basic and applied research (Galizi and Jaramillo, 2018).

In Zhao et al. paper, they described data sources as “the different *S. aureus* strain genomes were searched by the National Center for Biotechnology Information (NCBI) nucleotide database (http://www.ncbi.nlm.nih.gov/) with default parameters; then *S. aureus* CRISPR loci were searched by the CRISPR Finder server (E-value ≤ 0.001) (http://crispr.i2bc.paris-saclay.fr/Server/) (Last updated on May 9, 2017).” They found 22 strains of *S. aureus* contained one CRISPR locus, 14 strains of *S. aureus* contained 2 CRISPR loci, and the other 2 strains contained 3 and 4 CRISPR loci. We downloaded the genomes of 38 strains from NCBI nucleotide database (http://www.ncbi.nlm.nih.gov/) according to the GenBank ID they provided and then searched the CRISPR loci by CRISPR Finder. However, we found different results from their article description. As shown in Figure 1, only 5 strains were detected carrying the confirmed CRISPR locus, three of which contained one CRISPR locus, and the other 2 strains contained 2 and 3 CRISPR loci. Especially, the last strain, MSHR1132, is not a strain of *S. aureus*, but *Staphylococcus argenteus*. *S. argenteus* was given status as a separate species distinct from *S. aureus* in 2015 (Tong et al., 2015). According to the confirmed CRISPR locus, Cas proteins were searched by CRISPRCas Finder. We found all 4 strains of *S. aureus* contained Cas3 and only *S. aureus* 08BA02176 contained diverse Cas proteins that belongs to subtype III-A.

**Figure 1:**
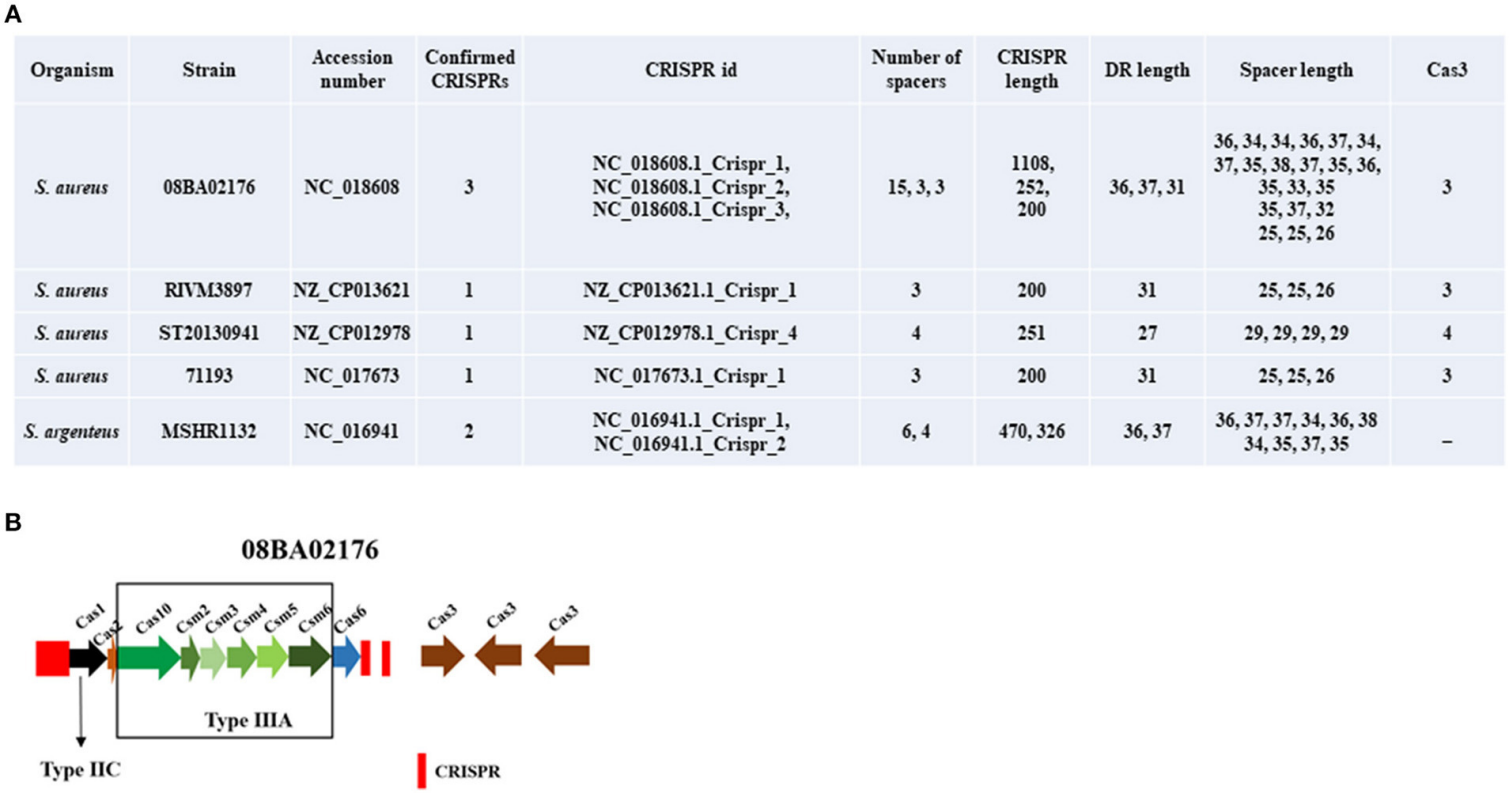
The features of confirmed CRISPR loci. **(A)** The statistical table of 8 CRISPR loci of strains. **(B)** The distribution of CRISPR and *cas* genes in *S. aureus* 08BA02176.

We found that the confirmed CRISPR loci searched by CRISPR Finder according to genomes from NCBI are different from the information in CRISPR database, although the CRISPR Finder and the CRISPR database are connected to each other. So, we should be cautious of the data sources when we use online database for analysis.

In summary, bioinformatics of CRISPR study would provide new ideas on the phylogenetic distribution and potential role of CRISPR-Cas systems in shaping the *S. aureus* accessory genome and antibiotic resistance elements.

## Author Contributions

TM and HY designed the study. TM and JL analyzed data and wrote the paper. GD offered some good advice. All authors read and approved the final manuscript.

### Conflict of Interest Statement

The authors declare that the research was conducted in the absence of any commercial or financial relationships that could be construed as a potential conflict of interest.
